# Dose-resolved serial synchrotron and XFEL structures of radiation-sensitive metalloproteins

**DOI:** 10.1107/S2052252519003956

**Published:** 2019-05-03

**Authors:** Ali Ebrahim, Tadeo Moreno-Chicano, Martin V. Appleby, Amanda K. Chaplin, John H. Beale, Darren A. Sherrell, Helen M. E. Duyvesteyn, Shigeki Owada, Kensuke Tono, Hiroshi Sugimoto, Richard W. Strange, Jonathan A. R. Worrall, Danny Axford, Robin L. Owen, Michael A. Hough

**Affiliations:** a School of Biological Sciences, University of Essex, Wivenhoe Park, Colchester CO4 3SQ, UK; b Diamond Light Source, Harwell Science and Innovation Campus, Didcot, Oxfordshire OX11 0DE, UK; c Division of Structural Biology (STRUBI), The Henry Wellcome Building for Genomic Medicine, University of Oxford, Roosevelt Drive, Oxford, Oxfordshire OX3 7BN, UK; d RIKEN SPring-8 Center, 1-1-1 Kouto, Sayo, Hyogo 679-5148, Japan; e Japan Synchrotron Radiation Research Institute, 1-1-1 Kouto, Sayo, Hyogo 679-5198, Japan

**Keywords:** XFELs, microcrystals, serial femtosecond crystallography, serial synchrotron crystallography, serial millisecond crystallography, fixed targets, heme peroxidase, metalloproteins, radiation damage

## Abstract

An approach is demonstrated to obtain, in a sample- and time-efficient manner, multiple dose-resolved crystal structures from room-temperature protein microcrystals using identical fixed-target supports at both synchrotrons and X-ray free-electron lasers (XFELs). This approach allows direct comparison of dose-resolved serial synchrotron and damage-free XFEL serial femtosecond crystallography structures of radiation-sensitive proteins. Specifically, serial synchrotron structures of a heme peroxidase enzyme reveal that X-ray induced changes occur at far lower doses than those at which diffraction quality is compromised (the Garman limit), consistent with previous studies on the reduction of heme proteins by low X-ray doses. In these structures, a functionally relevant bond length is shown to vary rapidly as a function of absorbed dose, with all room-temperature synchrotron structures exhibiting linear deformation of the active site compared with the XFEL structure. It is demonstrated that extrapolation of dose-dependent synchrotron structures to zero dose can closely approximate the damage-free XFEL structure. This approach is widely applicable to any protein where the crystal structure is altered by the synchrotron X-ray beam and provides a solution to the urgent requirement to determine intact structures of such proteins in a high-throughput and accessible manner.

## Abstract 2

A method is described that enables both time- and sample-efficient X-ray crystallographic data collection from radiation-sensitive protein microcrystals and direct comparison of the resulting dose-resolved serial synchrotron and damage-free X-ray free-electron laser structures. It is demonstrated that real-space extrapolation from a synchrotron dose series reproduces key features of the damage-free structure obtained using an X-ray free-electron laser.

## 1. Introduction   

Enzymology and structural biology are highly dependent on the accurate three-dimensional models obtained by X-ray crystallography. Such structures provide insight into function and can form a basis for understanding how proteins interact with each other or with small molecules. Fundamentally, the structure obtained should be representative of the native state of the protein. However, macromolecular crystallography is typically carried out at cryogenic temperatures (100 K) to minimize radiation-damage-induced structural perturbation (Garman & Weik, 2017 ▸; Holton, 2009 ▸). There is an increasing recognition of the importance of determining structures at ambient or ‘room’ temperature so as to be more representative of the structures and dynamics adopted by proteins *in vivo* at physiological temperature (Keedy *et al.*, 2018 ▸, 2015 ▸; Fischer *et al.*, 2015 ▸; Weik & Colletier, 2010 ▸). A major challenge in conventional synchrotron-based X-ray crystallography, particularly at room temperature, is the extremely rapid onset of radiation damage, *i.e.* changes to the structure of the protein caused by the ionizing effects of the X-ray beam (Garman & Weik, 2017 ▸; Holton, 2009 ▸). The rapidity of site-specific radiation damage means it is present in differing levels of severity in almost all crystallographic datasets determined using synchrotron radiation, even if great care to avoid it is taken during data collection by minimizing the absorbed dose; the challenge becomes even greater if only microcrystals are available. When collecting data from microcrystals, microbeams of increased brilliance are required for optimal data collection, though the use of such beams comes with a concomitant increase in the rate of X-ray induced changes. It is critical that the site-specific effects of radiolysis are understood in detail and minimized in order to produce structures that are accurately representative of radiation-sensitive proteins *in vivo*. We note however that in some protein crystals that do not contain metals or other redox centres, radiation damage, while present, may cause little change to the observed structure.

It is estimated that approximately one-third of all proteins require a metal ion, with around half of all enzymes utilizing a metal for catalytic function (Waldron *et al.*, 2009 ▸). Heme enzymes catalyse many essential reactions in biology and understanding their structures throughout their reaction cycles is of high interest, prompting extensive efforts made to obtain ‘intact’ structures at high resolution (Chreifi *et al.*, 2016 ▸; Casadei *et al.*, 2014 ▸; Gumiero *et al.*, 2011 ▸; Moody & Raven, 2018 ▸). A major challenge is to obtain the higher valence states of these proteins, for example peroxidases (Fe^III^ resting state and Fe^IV^ intermediate states), as these states are phenomenally sensitive to reduction in synchrotron experiments caused by the presence of large numbers of solvated electrons or other radiolytically produced species, generated by the interaction of X-rays with the crystal (Kekilli *et al.*, 2017 ▸; Beitlich *et al.*, 2007 ▸; Denisov *et al.*, 2007 ▸). This site-specific damage is known to occur at doses much lower than those typically required to collect a dataset (Garman & Weik, 2017 ▸; Holton, 2009 ▸). Structures of peroxidases and other redox-sensitive metalloproteins obtained from synchrotron X-ray crystallography, even at 100 K, are therefore likely to represent a superposition of resting and damaged states. This site-specific damage is an extremely pressing problem if mechanistic conclusions are to be drawn from the structures obtained.

In contrast, X-ray free-electron lasers (XFELs) promise damage and artefact-free crystallography, provided the pulse duration is short enough (Schlichting, 2015 ▸; Nass *et al.*, 2015 ▸; Lomb *et al.*, 2011 ▸). For serial femtosecond crystallography (SFX), data collection from each microcrystal can be completed before site-specific and global radiation damage occurs, but at the expense of longer term crystal destruction, such that a new crystal must be presented to every pulse. Radiation-induced changes have been detected in SFX data with pulse durations as short as 40 fs (Nass *et al.*, 2015 ▸) or 70 fs (Lomb *et al.*, 2011 ▸). In contrast, data measured with pulse durations of 10 fs or shorter are considered to be free of typical radiation-induced site-specific radiation damage (Halsted *et al.*, 2018 ▸; Andersson *et al.*, 2017 ▸). Direct comparison of XFEL and synchrotron structures of the same protein presents many challenges because of differences between these methods, such as crystal size, mosaicity, temperature, cryoprotection, crystallization conditions and resolution. The epitome of this being that almost all XFEL structures are obtained from tens of thousands of room-temperature microcrystals while most synchrotron structures are obtained from a single crystal held at 100 K.

We report a new method based on a highly efficient fixed-target silicon nitride chip system (Oghbaey *et al.*, 2016 ▸; Mueller *et al.*, 2015 ▸). This system allows for data to be measured at room temperature from microcrystals in the same manner using synchrotron or XFEL radiation. Our fixed-target approach enables time- and sample-efficient data collection by both SFX and serial synchrotron crystallography (SSX), and simultaneously minimizes any differences in structure by eliminating the experimental variables outlined above. It is also well suited to tracking functionally relevant changes in redox enzymes as X-ray generated solvated electrons drive the enzyme along the catalytic pathway in the crystal when exposed to the X-ray beam. Multiple serial structures (MSS) can be obtained from a set of crystals on a single fixed target, as sequential exposure events to each crystal are binned and processed as individual dose-dependent datasets. This is analogous to the multiple structures from one crystal (MSOX) approach (Horrell *et al.*, 2016 ▸, 2018 ▸), previously applied to the measurement of repeated datasets from the same exposed region of a single large crystal to produce a dose series. In comparison, our new approach exposes each crystal to the X-ray beam for only a few tens of milliseconds and is well suited for high-throughput structure solution from microcrystals held at room temperature, using XFEL or synchrotron radiation sources.

Herein, we have chosen to use an extracellular dye-type heme peroxidase found in *Streptomyces lividans* and referred to as DtpAa. Using DtpAa as the exemplar, we describe the application of our method of combined SFX and MSS experiments, though the method can be used for any redox-sensitive system. Starting in the catalytic resting state, our approach reveals multiple well resolved structural states of the enzyme, with a low-dose synchrotron MSS structural series showing clearly resolved changes to the active-site region of the enzyme within tens of milliseconds. Extrapolation of varying structural parameters to zero dose produced a close match to the damage-free structure determined using SFX. Thus, a low-dose series of synchrotron MSS is anchored by a damage-free SFX structure, both being determined using the same fixed-target serial sample-delivery system.

We present this approach as a general method to efficiently collect both SFX and SSX data under near-identical conditions, characterize subtle site-specific changes caused by X-rays in proteins and allow direct comparison of, and extrapolation to, damage-free XFEL structures from low-dose synchrotron models.

## 2. Materials and methods   

### 2.1. Sample preparation   

The SLI_2602 gene encoding DtpAa was amplified from the genomic DNA of *S. lividans* strain 1326 (*S. lividans* stock number 1326, John Innes Centre) by polymerase chain reaction (the primers used for amplification are reported in the Supporting information). The gene was subsequently cloned into the NdeI and HindIII sites of a pET28a vector (Novagen) to create an N-terminal His_6_-tagged construct (pET2602) for overexpression in *Escherichia coli*. The pET2602 vector was transformed into *E. coli* BL21 (DE3) cells. Overnight pre-cultures [low-salt Luria–Bertani (LB) medium; Melford] were successively used to inoculate 1.4 l of high-salt LB medium with 50 mg ml^−1^ kanamycin and were grown at 37°C, 180 rev min^−1^. At an OD_600_ of 1.0–1.2, 5-amino­levulinic acid (0.25 m*M* final concentration) and iron citrate (100 µ*M* final concentration) were added consecutively for their use as a heme precursor and iron supplement, respectively. Cultures were then induced by adding iso­propyl β-d-thio­galacto­pyran­oside (Melford) to a final concentration of 0.5 m*M*, and carbon monoxide gas was bubbled through the culture for 30–60 s. Flasks were then sealed and incubated for a further 18 h at 30°C and 100 rev min^−1^. Cells were harvested via centrifugation (10 000*g*, 10 min, 4°C) and the cell pellet resuspended in 50 m*M* Tris–HCl, 500 m*M* NaCl (Fisher) and 20 m*M* imidazole (Sigma) at pH 8 (buffer A). The resuspended cell suspension was lysed using an EmulsiFlex-C5 cell disrupter (Avestin) followed by centrifugation (22 000*g*, 30 min, 4°C). The clarified supernatant was loaded onto a 5 ml nickel–nitrilo­tri­acetic acid–sepharose column (GE Healthcare) equilibrated with buffer A and eluted by a linear imidazole gradient using buffer B (buffer A with 500 m*M* imidazole). The DtpAa peak eluting at approximately 30–40% buffer B was pooled and concentrated using a Centricon (VivaSpin) with a 10 kDa cut-off at 4°C followed by application to an S200 Sephadex column (GE Healthcare) equilibrated with 20 m*M* NaPi, 100 m*M* NaCl, pH 7. A major peak eluted consistent with a monomeric species with fractions assessed by SDS–PAGE then concentrated and stored at −20°C. DtpAa concentrations were determined by UV–vis spectroscopy (Varian Cary 60 UV–vis spectrophotometer) using an extinction coefficient at 280 nm of 46 075 *M* ^−1^ cm^−1^.

Microcrystals were grown in batch (typically 0.4–0.5 ml total volume) by mixing in a 1:1 ratio a 6.5 mg ml^−1^ DtpAa protein solution with a precipitant solution containing 20% PEG 6000, 100 m*M* HEPES pH 7.0, to typical dimensions of 15 µm. Silicon nitride fixed-target chips with either 7, 12, 14 or 37 µm apertures at their narrowest opening and a nominal capacity of 25 600 crystals were loaded as previously described (Ebrahim *et al.*, 2019 ▸), with an identical loading protocol used both at Diamond and the SPring-8 Ångstrom Free Electron Laser (SACLA) XFEL. In brief, chips were loaded with 100–200 µl of microcrystal suspension within a humidity enclosure (Solo Containment, Cheshire, England) and sealed between two layers of 6 µm thick Mylar.

### 2.2. Data collection and fixed-target motion   

SFX data were measured at SACLA beamline BL2 EH3 using an X-ray energy of 10.0 keV, a pulse length 10 fs and a repetition rate of 30 Hz, with the beam attenuated to 13% of full flux. Chips were translated within the interval between X-ray pulses, ensuring that the chip had stopped at the centre of each crystal position (the centre of the aperture) and was exposed only once to X-rays, before moving to the next position during the next pulse interval. Data were typically collected from all 25 600 positions on a chip in 14 min using the SACLA MPCCD detector (Kameshima *et al.*, 2014 ▸), with experiments performed in a helium chamber to minimize air scatter. A modified custom entry port to the helium chamber permitted rapid exchange of chips, meaning that measurement from all positions with subsequent sample exchange and alignment interval of <5 min between data collections allowed a sustained data-collection rate of just over 3 chips per hour. While sufficient data for structure solution and refinement were obtained from crystals mounted on only 2 chips (*ca* 13 000 hits), for the structure described here data were collected from a total of 11 chips, still in under 4 h of beam time, in order to increase the redundancy of the data and the quality of the maps obtained.

**Figure 1:**
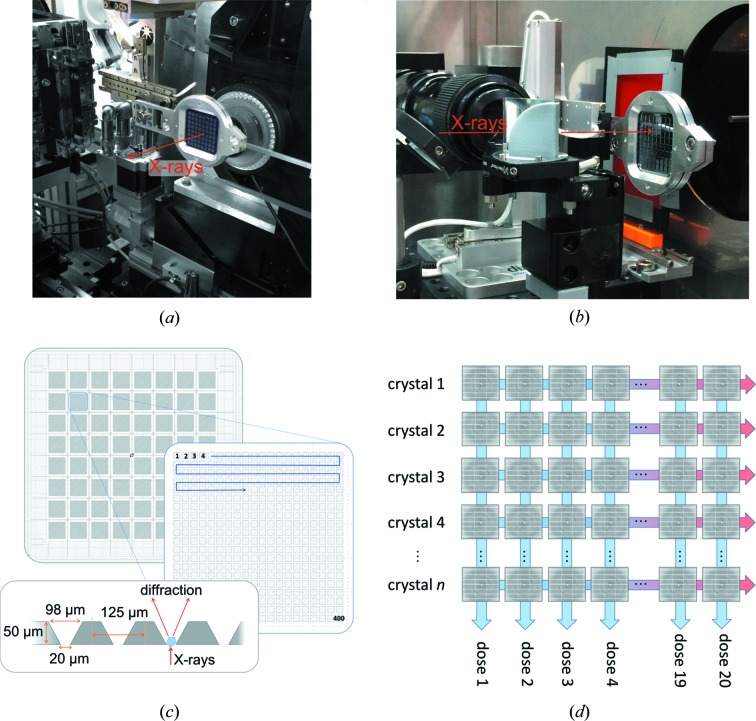
Fixed-target instrumentation in place at (*a*) beamline I24, Diamond Light Source and (*b*) beamline BL2 EH3, SACLA. (*c*) Schematic of fixed target used showing layout of 8 × 8 ‘city blocks’, each comprising 20 × 20 apertures. Shown is a zoomed-in view of a single city block with motion path followed and chip cross-section. (*d*) Formation of dose-resolved datasets by collecting multiple images at each chip aperture. For XFEL data collection, only a single dose point is recorded at each position.

Data collection at beamline I24, Diamond Light Source was carried out using an unattenuated X-ray beam of energy 12.8 keV and a Pilatus3 6M detector in shutterless mode. To form a dose-dependent series of DtpAa structures, 5 (MSS-1) and 10 (MSS-2) sequential diffraction patterns were measured at each crystal position each with an exposure time of 10 ms and subsequently binned into one dataset per dose interval (Fig. 1 ▸). The series of exposures at each chip position was individually triggered via a Keysight 33500B signal generator which was itself triggered by a DeltaTau Geobrick LV-IMS-II stage controller when each desired crystal position had been attained. The X-ray shutter was not closed between apertures on a chip and remained open for the duration of the experiment. X-ray fluxes were measured using a silicon PIN diode as previously described (Owen *et al.*, 2006 ▸) and were 3.2 × 10^12^ and 3.0 × 10^12^ photons s^−1^ for MSS-1 and MSS-2, respectively. The corresponding beamsizes (measured using a knife-edge scan) were 7 × 7 and 9 × 8 µm, respectively. Absorbed doses were estimated using *RADDOSE*-3*D* (Zeldin *et al.*, 2013 ▸), with dose increments corresponding to the total dose accumulated within the exposure time of the first image, and are detailed in Table S1 in the Supporting information. We note that crystals will be subjected to a small additional absorbed dose during deceleration of the stages prior to the time when the detector starts recording the first diffraction image. While challenging to accurately determine, we estimate that an upper bound for this dose is ∼3 kGy. These experiments were carried out using the same fixed-target chips and translation system as used at SACLA. Details of the datasets are given in Table S1.

### 2.3. Data analysis   

For data measured at SACLA, initial hit finding at the beamline was carried out in *CHEETAH* (Barty *et al.*, 2014 ▸). Peak-finding, integration and merging were all performed in *CrystFEL* (White *et al.*, 2016 ▸). Data from Diamond beamline I24 were indexed using *DIALS* (version 1.8.5) (Winter *et al.*, 2018 ▸) with subsequent scaling and merging performed using *PRIME* (Uervirojnangkoorn *et al.*, 2015 ▸). MSS data from beamline I24 consisted of cbf image files numbered sequentially. These were binned into dose points using a simple partitioning script. Multiple lattices were allowed during indexing.

In both cases resolution limits were assessed using CC_1/2_ and *R*_split_ parameters (White *et al.*, 2013 ▸, 2016 ▸) together with behaviour in refinement. Structures were solved by molecular replacement using a starting model obtained from a small number of larger DtpAa crystals mounted between two layers of thin film (Axford *et al.*, 2012 ▸; Doak *et al.*, 2018 ▸) and used to obtain rotation wedges. Water molecules were removed from this model prior to refinement. Structures were refined initially using *REFMAC*5 (Murshudov *et al.*, 2011 ▸) within the *CCP*4 suite (Winn *et al.*, 2011 ▸) and later in *PHENIX* (Adams *et al.*, 2010 ▸) and rebuilt between refinement cycles using *Coot* (Emsley *et al.*, 2010 ▸). Atoms not well supported by electron density (primarily surface side chains) were deleted from the model. Validation was performed using *MolProbity* (Richardson *et al.*, 2018 ▸), *QCCheck* and tools within *Coot* and *PHENIX*. Estimates of bond-length error were calculated from the coordinate diffraction precision index as described (Gurusaran *et al.*, 2014 ▸) using the online diffraction precision indicator (DPI) server (Kumar *et al.*, 2015 ▸).

## 3. Results   

### 3.1. Sample- and time-efficient serial data collection at synchrotron microfocus and XFEL beamlines using silicon nitride fixed-target chips   

We used high-capacity silicon nitride fixed targets or ‘chips’ each containing 25 600 apertures based on those described previously (Oghbaey *et al.*, 2016 ▸) to hold the microcrystals used to determine room-temperature serial crystallography structures of DtpAa. Importantly, this sample-delivery system was used in a near-identical manner for both the SFX and the MSS experiments, (Fig. 1 ▸), allowing for a direct comparison of the resulting structures. Typically hit rates (we define hit rate as the percentage of frames collected that could be indexed) of ∼30% were achieved on each chip allowing structures to be determined in a highly time- and sample-efficient manner. The volume of microcrystal suspension required per chip was typically 100–200 µl. A schematic of the chip setup and methodological approach is shown in Fig. 1 ▸.

### 3.2. Damage-free DtpAa structure using serial femtosecond crystallography   

**Figure 2:**
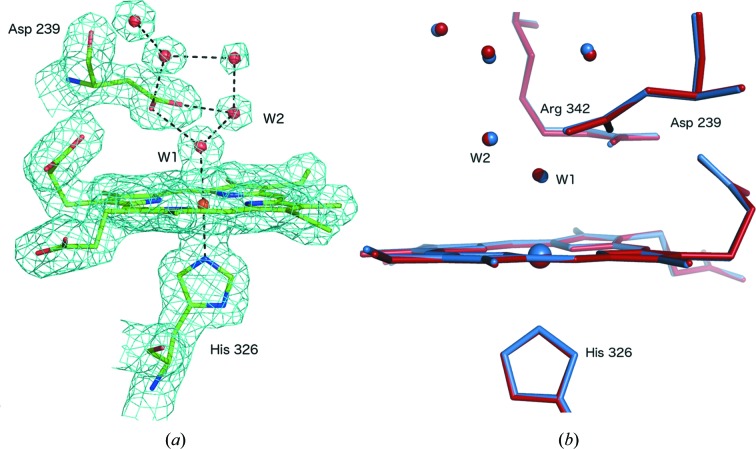
(*a*) 2*F*_o_–*F*_c_ electron-density map contoured at 1σ for the damage-free SFX structure of DtpAa at 1.88 Å resolution, showing the clear and well resolved water network within the heme pocket. Water molecules interact extensively with the pocket residue Asp239 as well as with Arg369 (omitted for clarity). (*b*) Superposition of the SFX structure (blue) with the 32.8 kGy SSX structure (red). Small changes to the heme-pocket water network are apparent even at this low dose.

To produce an ‘anchor’ structure of DtpAa, *i.e*. resting state ferric, free of any effects of the X-ray beam on the structure, we used the SACLA XFEL (Ishikawa *et al.*, 2012 ▸) beamline BL2 EH3 to perform SFX with an X-ray energy of 10 keV, a pulse length of 10 fs with a 1.25 × 1.34 µm beam and a pulse energy of 289 µJ pulse^−1^. The chip was translated between apertures in the 33 ms separating the 30 Hz XFEL pulses, with a single image recorded at each position. The SFX structure was determined to a resolution of 1.88 Å from a total of 72 615 indexed and merged diffraction patterns [Fig. 2 ▸(*a*), Table S1]. The overall structure reveals a ferredoxin-like fold typical of dye decolourizing peroxidases (Sugano, 2009 ▸) with two DtpAa monomers in the crystallographic asymmetric unit. The structure was of high quality (Table S1) and refined to an *R*_work_ and *R*_free_ of 13.2% and 16.7%, respectively. The refined model exhibited a mean-determined *B* factor of 34.7 Å^2^.

The heme Fe is six-coordinate with residue His326 acting as the proximal ligand with an Fe—N bond length of 2.19 Å (we note here that monomer B appears to be inactive and is consequently not discussed further). The distal heme coordination site is occupied by a well defined, full occupancy water molecule (W1), bound to the Fe at a distance of 2.40 Å. A number of further, well defined water molecules occupy the remainder of the heme distal pocket [Fig. 2 ▸(*a*)]. W1 is hydrogen bonded to a second water, W2, at a distance of 2.68 Å and also interacts with the charged side chains of Asp239 (2.92 Å) and Arg342 (2.74 Å). Interestingly, the side chains of these two amino acids are only 3.13 Å apart (Arg N_η1_ to Asp O_δ2_) suggesting a charge-based interaction.

### 3.3. DtpAa structures from serial synchrotron crystallography   

Serial synchrotron crystallography was carried out at Diamond Light Source beamline I24 at an X-ray energy of 12.8 keV using the same chip and translation system as used for SFX at SACLA. The beam size and flux were measured immediately prior to each experiment, see Materials and methods[Sec sec2] for details, with approximate values of 7 × 7 µm and 3.1 × 10^12^ photons s^−1^. Following each translation of the chip to bring a fresh aperture/crystal into the beam, a series of 10 ms exposures were recorded using the PILATUS3 detector in shutterless mode. This allowed multiple successive snapshots of the same microcrystal within 100 ms. Following exposure of a crystal to the X-ray beam, the chip was translated to the next aperture position and the process repeated [shown schematically in Fig. 1 ▸(*d*)]. Using this approach, the total experimental time per fully loaded chip for ten dose points is 45 min, but the total exposure (and hence the absorbed dose) of any individual microcrystal is low and multiple time- (dose-) resolved structures are obtained from a single fixed target. We note here that the 10 ms minimum exposure time was imposed by the maximum frame rate of the current detector available (PILATUS3 6M) and not by limitations arising from the fixed-target movement or synchronization of the target and the X-ray beam. Diffraction images were indexed and integrated independently using *DIALS* (dials.stills_process) (Winter *et al.*, 2018 ▸) with a simple image-binning procedure used to assign the resulting data to dose bins [Fig. 1 ▸(*d*)]. Data within each dose bin were then scaled and merged together using *PRIME* (Uervirojnangkoorn *et al.*, 2015 ▸) to form dose-resolved datasets. Using this approach, a complete dataset for each X-ray dose was formed and the corresponding structure refined using the methods described above. The scaling and refinement statistics for each structure are given in Table S1. We first describe an MSS experiment series comprising five dose points with a dose increment of 32.8 kGy (MSS1). An increase in unit-cell volume and trends in scaling statistics clearly indicate the onset of global radiation damage resulting from disorder in the crystalline lattice as dose is accumulated (Fig. S2). The initial resolution was 1.78 Å with only a limited loss of diffracting power/resolution during the 50 ms of total exposure for each microcrystal. Dataset 1 of this series (MSS1-ds1, 32.8 kGy) reveals a six-coordinate heme with a slightly lengthened Fe—O bond at 2.48 Å compared with the SFX structure [Fig. 2 ▸(*b*)]. A superposition of MSS1-ds1 with the SFX structure is shown in Fig. 2 ▸(*b*). With increasing dose, distinct changes occur around the heme pocket consistent with reduction of the heme iron by X-ray generated solvated photoelectrons (Beitlich *et al.*, 2007 ▸; Kekilli *et al.*, 2017 ▸). In MSS1-ds2, the Fe—O bond is 2.70 Å with this continuing to lengthen until the last dataset (MSS1-ds5, 164.0 kGy) where it reaches a value of 2.97 Å (Table S3).

**Figure 3:**
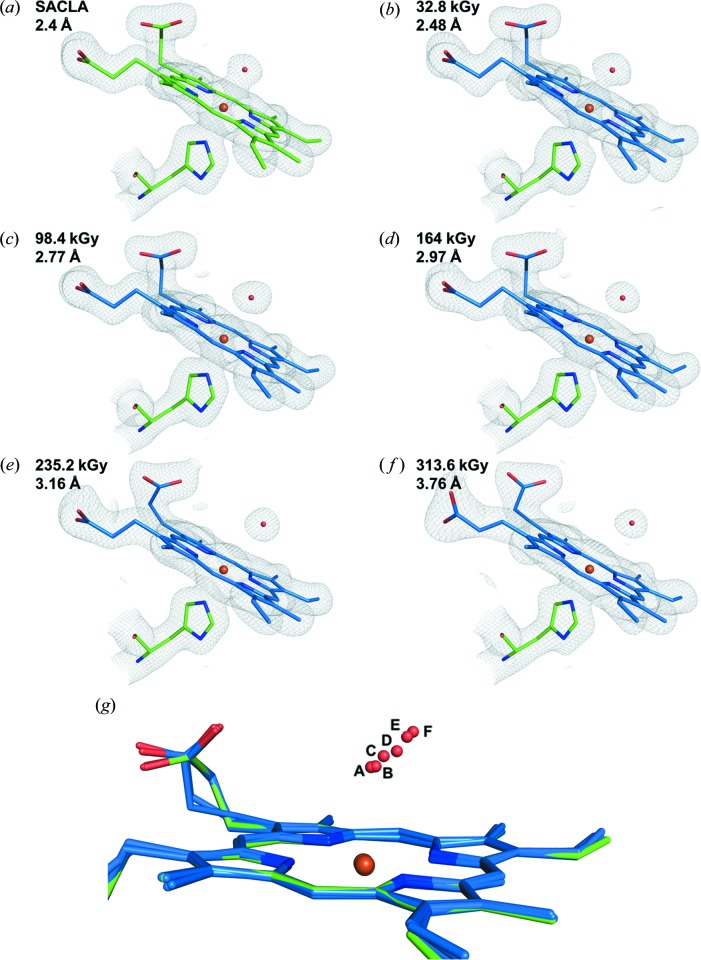
2*F*_o_–*F*_c_ electron-density maps contoured at 1σ for the heme environment of DtpAa in (*a*) the SFX dataset from SACLA and (*b*–*f*) selected structures from the two MSS series. (*g*) Superposition of selected structures revealing the dose-dependent migration of the water molecule W1 away from the heme Fe. The SFX structure is shown in green with MSS in blue.

In order to provide additional dose points and obtain higher dose SSX structures, a second MSS series was measured with an increased incremental dose value (MSS2). In this case, 10 × 10 ms exposures were measured per crystal position (Table S1 and Fig. S3) with a dose interval of 39.2 kGy. The initial dataset was refined to a resolution of 1.70 Å with the resolution remaining as high as 1.93 Å by dose point 6. After this point (60 ms exposure) the resolution limit decayed, with structure refinement only carried out to 2.18 Å resolution by the last dataset (MSS2-ds8). For comparison, dataset 10 reached only 2.7 Å resolution. In this series the first dataset (MSS2-ds1), associated with a dose of 39.2 kGy, exhibited a Fe–W1 distance of 2.50 Å. This distance increased in successive dose point structures, reaching 2.64 Å in ds3, 2.91 Å in MSS2-ds5 and 3.76 Å in MSS2-ds8 (Fig. 3 ▸, Tables S1 and S2). Additional structural changes were evident in the heme pocket, with rearrangement of water structures and a flip of a heme propionate as dose was accumulated (Figs. S4 and S5).

**Figure 4:**
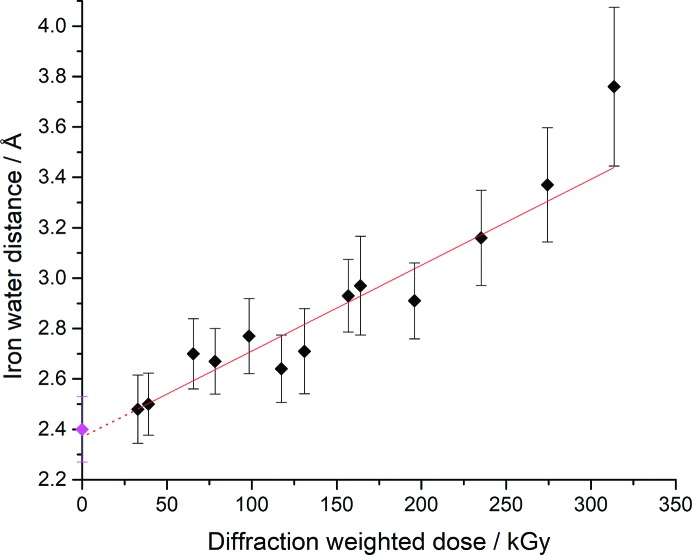
Plot of Fe–W1 distance as a function of X-ray dose from the two measured MSS series. The SFX structure determined using SACLA is plotted as the zero-dose point (magenta). The elongation of the bond length with dose is well fitted by a linear function (red line). The deviation at higher doses is associated with a dissociation of W1 from the immediate vicinity of the heme Fe. The extrapolation to zero dose (dashed red line) gives a value of 2.37 (±0.05) Å which is very close to the 2.40 (±0.13) Å value of this parameter in the SFX structure. Error bars shown are the estimated standard uncertainty in bond length obtained from the DPI value of the Fe and W1 atoms (see Materials and methods[Sec sec2]).

The Fe–O distance in all structures from both MSS series is plotted in Fig. 4 ▸ and migration of the water away from the heme Fe is shown in Fig. 3 ▸(*g*).

It is of considerable interest to compare low-dose synchrotron structures and damage-free XFEL structures determined under near-identical experimental conditions, and to explore if dose-series data may be used to extrapolate back to the ‘native’ state present prior to X-ray exposure, a so-called ‘zero-dose extrapolation’. This approach is analogous to the zero-dose extrapolation of diffraction intensities within conventional single-crystal datasets that has been described previously (Diederichs *et al.*, 2003 ▸; Diederichs, 2006 ▸; Diederichs & Junk, 2009 ▸). In this way, the SFX structure provides a starting point from which synchrotron datasets (inevitably incurring radiation damage and consequent structural change) may be interpreted. A vivid example is shown in a plot of the Fe–W1 distance in the SFX ‘anchor’ structure and both MSS series, Fig. 4 ▸. A near-linear relationship is observed, demonstrating that water migration away from the Fe is dose-dependent under the conditions used. A linear fit to the data yields an intercept (*i.e.* extrapolated to zero dose) of 2.37 Å, which is very close to the value in the SFX structure (2.40 Å) at comparable resolution and within the experimental error for this bond length in the room-temperature structures. The SFX and MSS datasets were deposited in the Protein Data Bank with accession codes as indicated in the supplementary tables in the Supporting information.

## 4. Discussion   

To our knowledge, this is the first reported method for directly comparing dose-resolved serial synchrotron and XFEL structures of radiation-sensitive metalloproteins using the same microcrystal preparations and sample-delivery system. The resolutions achieved with each X-ray source are comparable (1.88 Å SFX and 1.70 Å SSX) allowing the direct comparison of structural features. The SSX data collection allowed sequences of 5–10 MSS dose points to be measured in a time of tens to hundreds of milliseconds per microcrystal. The effective resolution remained high for a substantial proportion of each series. Interestingly, the MSS structures of DtpAa showed well resolved water molecules (Figs. 2 ▸ and 3 ▸) in all structures, indicating that the progression of reactions within the exposed crystal volume is relatively uniform.

Determining a sequence of dose-dependent structures from the same microcrystals allows subtle and relatively rapidly occurring structural changes to be resolved. In the case of DtpAa, an elongation of the Fe—water bond and eventual bond breakage were observed during tens of milliseconds of exposure to an intense microfocus X-ray beam. By obtaining MSS throughout the process, sufficient data points were recorded in order to be able to fit a function with confidence and allow a zero-dose extrapolation to be made. This provided a close approximation to the structure determined by SFX, providing an alternative approach to obtain a good approximation of the ‘gold standard’ damage-free structure obtained using an XFEL.

### 4.1. How close can we get to the damage-free enzyme structure using synchrotron radiation?   

Despite a relatively low absorbed dose of 32.8 kGy in the MSS1-ds1 dataset, the structure is not identical to that determined by SFX [Fig. 2 ▸(*b*)]. Notably, the iron—water bond in MSS1-ds1, the shortest out of all the SSX structures, is elongated compared with the SFX structure. A simple linear fit of the plot of iron—water bond length as a function of dose allowed an extrapolation of the SSX data to zero dose (*y*-axis intercept), yielding a comparable distance to that observed in the SFX structure (Fig. 4 ▸).

While not the main focus of this report, the MSS series we present reveal a number of structural states populated during the initial response of DtpAa to X-rays. The elongation of the iron—water bond is consistent with Fe^III^ to Fe^II^ reduction. This reduction is consistent with the generation of solvated electrons by the interaction of X-rays with solvent molecules in the crystal (Kwon *et al.*, 2017 ▸; Moody & Raven, 2018 ▸). In contrast to the situation at 100 K, where X-ray generated radicals are largely immobilized, room-temperature reactions that involve mass transport may allow such radicals to contribute to the structural changes that our methods allow to be resolved. The relevance of these structures to the function of this class of enzymes will be explored in detail elsewhere.

An additional advantage of our approach is that the same methodology and sample-delivery system is used at synchrotron and XFEL sources/beamlines. This allows for an effective comparison of the structures produced by each X-ray source, allowing the use of comparable crystal sizes, temperatures and sample-delivery methods, factors that might otherwise cause structural heterogeneity.

In summary, we have shown that microcrystals loaded into fixed-target silicon nitride chips can be efficiently employed for data collection at both synchrotron and XFEL sources, allowing near-identical conditions for experiments. Using this technology, we have characterized subtle site-specific changes caused by X-rays in proteins, and directly compared low-dose synchrotron models with, and extrapolation to, damage-free SFX structures. Our method has the potential to be applied to a wide range of enzymes and other proteins especially those that are highly sensitive to radiation damage, including the characterization of electron-driven mechanistic steps in detail through a dose series such as redox reactions in redox metalloenzymes. On a practical level, our approach can be used to extract functionally relevant features of damage-free SFX structures (which require access to scarce beam time at XFELs), reconstructed from extrapolation of MSS determined at multiple low-dose points. Notably, the time interval per MSS structure will be reduced by at least an order of magnitude with upcoming advances in detectors and synchrotron brilliance.

## Supplementary Material

Supplementary tables and figures. DOI: 10.1107/S2052252519003956/ec5012sup1.pdf

PDB reference: Dye-type peroxidase Aa from *Streptomyces lividans*, MSS 78.4 kGy structure, 6i8p

PDB reference: 274.4 kGy structure, 6q3e

PDB reference: 235.2 kGy structure, 6q3d

PDB reference: 164 kGy structure, 6i8k

PDB reference: 98.4 kGy structure, 6i8i

PDB reference: 32.8 kGy structure, 6i7z

PDB reference: 39.2 kGy structure, 6i8o

PDB reference: 196 kGy structure, 6q34

PDB reference: 117.6 kGy structure, 6i8q

PDB reference: 65.6 kGy structure, 6i8e

PDB reference: 156.8 kGy structure, 6q31

PDB reference: 131.2 kGy structure, 6i8j

PDB reference: 313.6 kGy structure, 6ibn

PDB reference: SFX structure of damage-free ferric state of dye-type peroxidase Aa from *Streptomyces lividans*, 6i43
