# Supplementary material for: Dose-resolved serial synchrotron and XFEL structures of radiation-sensitive metalloproteins
Source: IUCrJ. 2019 May 3;6(Pt 4):543–51. doi: 10.1107/S2052252519003956 (PMC6608622; doi:10.1107/S2052252519003956)
Supplement: Supplementary file 1 [file m-06-00543-sup1.pdf]

IUCrJ

Volume 6 (2019)

Supporting information for article:

Dose-resolved serial synchrotron and XFEL structures of radiation-sensitive metalloproteins

Ali Ebrahim, Tadeo Moreno-Chicano, Martin V. Appleby, Amanda K. Chaplin, John H. Beale, Darren A. Sherrell, Helen M. E. Duyvesteyn, Shigeki Owada, Kensuke Tono, Hiroshi Sugimoto, Richard W. Strange, Jonathan A. R. Worrall, Danny Axford, Robin L. Owen and Michael A. Hough

Table S1 Data collection and processing statistics for the SFX structure and MSS series 1.

All structures are in space group $P2_1$.

Dataset	SACLA SFX	MSS1-ds1	MSS1-ds2	MSS1-ds3	MSS1-ds4	MSS1-ds5
Data Collection						
Cumulative dose / kGy	N/A	32.8	65.6	98.4	131.2	164.0
Number of integrated frames	73281	9751	9833	10002	9801	9243
Number of images used*	72615	8596	8608	8700	8342	7787
Data processing						
Space Group	$P2_1$					
Cell dimensions / Å/deg.	a=72.72, b=68.18, c=74.62, beta=105.58	a = 72.95 (0.33) b = 68.30 (0.13)	a = 72.96 (0.35) b = 68.30 (0.14)	a = 72.97 (0.36) b = 68.29 (0.15)	a = 72.99 (0.39) b = 68.29 (0.17)	a = 73.01 (0.42) b = 68.28 (0.20)

		c = 74.78 (0.43)<br><br>β = 105.65 (0.21)	c = 74.79 (0.45)<br><br>β = 105.68 (0.22)	c = 74.82 (0.46)<br><br>β = 105.72 (0.25)	c = 74.86 (0.49)<br><br>β = 105.76 (0.28)	c = 74.92 (0.54)<br><br>β = 105.79 (0.34)
Resolution / Å	41.46-1.88	44.64 - 1.78<br>(1.81 - 1.78)	44.63 - 1.78<br>(1.81 - 1.78)	44.62 - 1.83<br>(1.86 - 1.83)	44.63 - 1.90<br>(1.93 - 1.90)	44.64 - 1.98<br>(2.01 - 1.98)
Number of reflections	57351	67891	67896	62552	55954	49513
R_{split}	7.22 (58.29)	18.92 (47.72)	18.03 (51.62)	16.97 (56.25)	16.40 (61.71)	16.73 (62.72)
$CC_{1/2}$	99.27 (0.722)	94.43 (67.71)	95.34 (67.20)	95.99 (66.77)	96.63 (59.07)	96.94 (60.18)
Multiplicity	380.6 (212.6)	27.12 (11.36)	28.36 (10.48)	32.21 (10.79)	34.48 (10.19)	35.48 (9.39)
Completeness (%)	100 (100)	100 (99.9)	100 (99.9)	100 (99.9)	100 (99.8)	100 (99.8)
Refinement						
Number of reflections	57312	67891	67896	62551	55953	49512
Rwork/Rfree	0.132/0.167	0.1647 / 0.2032	0.1662 / 0.2114	0.1668 / 0.2142	0.1670 / 0.2120	0.1720 / 0.2322

RMSD bond lengths (Å)	0.009	0.006	0.006	0.006	0.007	0.007
RMSD bond angles (deg)	0.92	0.81	0.82	0.84	0.87	0.89
Ramachandran plot						
Most favoured (%)	98.5	98.3	98.2	98.1	97.5	96.9
Allowed (%)	1.50	1.67	1.81	1.81	2.50	3.06
PDB accession code	6I43	6I7Z	6I8E	6I8I	6I8J	6I8K

* Number of merged patterns in CrystFEL.

Table S2 Data collection and processing statistics for MSS series 2.

All structures are in space group $P2_1$.

Dataset	MSS2-1	MSS2-2	MSS2-3	MSS2-4	MSS2-5	MSS2-6	MSS2-7	MSS2-8
Data Collection								
Cumulative dose / kGy	39.2	78.4	117.6	156.8	196	235.2	274.4	313.6
Number of integrated frames	15294	14719	14032	13307	12464	11524	10058	8492
Number of images used	13024	12513	11889	11121	10210	9070	7678	6221
Data processing								
Cell dimensions / Å	a = 72.99 (0.54)<br>b = 68.36 (0.31)<br>c = 74.95 (0.61)	a = 73.01 (0.55)<br>b = 68.36 (0.31)<br>c = 74.94 (0.61)	a = 73.03 (0.55)<br>b = 68.36 (0.32)<br>c = 74.94 (0.62)	a = 73.04 (0.56)<br>b = 68.37 (0.33)<br>c = 74.97 (0.63)	a = 73.06 (0.59)<br>b = 68.37 (0.35)<br>c = 75.03 (0.66)	a = 73.07 (0.63)<br>b = 68.38 (0.37)<br>c = 75.08 (0.70)	a = 73.07 (0.67)<br>b = 68.38 (0.40)<br>c = 75.10 (0.74)	a = 73.07 (0.73)<br>b = 68.39 (0.45)<br>c = 75.06 (0.80)

	β = 105.63 (0.43)	β = 105.67 (0.44)	β = 105.72 (0.44)	β = 105.76 (0.47)	β = 105.81 (0.52)	β = 105.84 (0.54)	β = 105.85 (0.60)	β = 105.82 (0.65)
Resolution / Å	44.71 - 1.70 (1.73 - 1.70)	44.69 - 1.73 (1.76 - 1.73)	44.67 - 1.74 (1.77 - 1.74)	44.68 - 1.78 (1.81 - 1.78)	44.70 - 1.82 (1.85 - 1.82)	44.71 - 1.93 (1.96 - 1.93)	44.72 - 2.03 (2.07 - 2.03)	44.71 - 2.18 (2.22 - 2.18)
Number of reflections	78148	74214	72957	68910	64459	54263	46140	37330
R_{split}	18.24 (73.81)	18.31 (71.41)	18.15 (73.88)	18.05 (71.35)	17.38 (74.98)	17.17 (79.11)	17.90 (78.30)	18.79 (74.51)
$CC_{1/2}$	95.78 (49.99)	95.87 (49.15)	95.09 (51.04)	95.59 (52.06)	96.56 (50.64)	96.63 (54.40)	96.92 (50.62)	96.87 (53.87)
Multiplicity	52.99 (12.44)	51.36 (13.38)	48.90 (11.86)	47.42 (11.65)	45.97 (10.87)	46.51 (12.73)	44.54 (11.42)	41.36 (11.78)
Completeness (%)	100 (99.9)	100 (100)	100 (99.8)	100 (99.8)	100 (99.5)	100 (99.8)	100 (99.6)	100 (99.5)
Refinement								
Number of reflections	78148	74203	72955	68181	63833	53667	46136	37329
Rwork/Rfree	0.1805 / 0.2346	0.1774 / 0.2337	0.1780 / 0.2304	0.1784 / 0.2321	0.1798 / 0.2359	0.1803 / 0.2355	0.1816 / 0.2492	0.1778 / 0.2555

RMSD bond lengths (Å)	0.007	0.007	0.007	0.007	0.007	0.007	0.008	0.008
RMSD bond angles (deg)	0.85	0.84	0.85	0.85	0.86	0.91	0.94	0.98
Ramachandran plot								
Most favoured (%)	97.91	98.05	98.05	97.08	97.22	96.66	96.52	94.44
Allowed (%)	2.09	1.95	1.95	2.78	2.78	3.34	3.34	5.15
PDB accession code	6I8O	6I8P	6I8Q	6Q31	6Q34	6Q3D	6Q3E	6IBN

Table S3 **(a)** Heme site parameters for SFX and MSS-1 datasets.

Structure	SFX	MSS1-1	MSS2-2	MSS2-3	MSS2-4	MSS2-5
Resolution (Å)	1.88	1.78	1.78	1.83	1.90	1.98
Fe-His (Å)	2.19	2.24	2.25	2.29	2.25	2.23
Fe-W1 (Å)	2.40	2.48	2.70	2.77	2.71	2.97
PDB accession code	6I43	6I7Z	6I8E	6I8I	6I8J	6I8K

(b) Heme site parameters for MSS-2 datasets.

Structure	SFX	MSS2-1	MSS2-2	MSS2-3	MSS2-4	MSS2-5	MSS2-6	MSS2-7	MSS2-8
Resolution (Å)	1.88	1.70	1.73	1.74	1.78	1.82	1.93	2.03	2.18
Fe-His (Å)	2.19	2.27	2.27	2.31	2.30	2.28	2.22	2.13	2.23
Fe-W1 (Å)	2.40	2.50	2.67	2.64	2.93	2.91	3.16	3.37	3.76
PDB accession code	6I43	6I8O	6I8P	6I8Q	6Q31	6Q34	6Q3D	6Q3E	6IBN

2602-F
5'-CTAACATATGGACCCGGCCGGTGCCGACGCG-3'

2602-R
5'-CCGAAGCTTTTACGCCTCCTTGCCGAACAGCG-3'

Figure S1 DtpAa amplification primers.

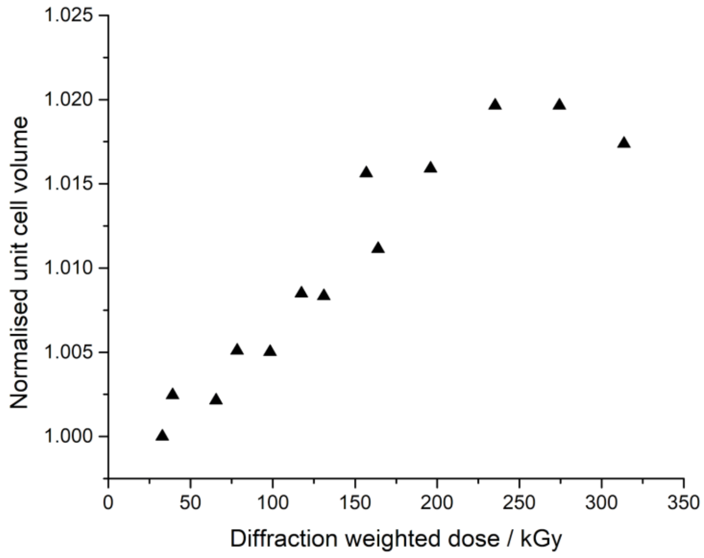

Figure S2 Unit cell volume vs. absorbed dose for MSS data collected at Diamond beamline I24.

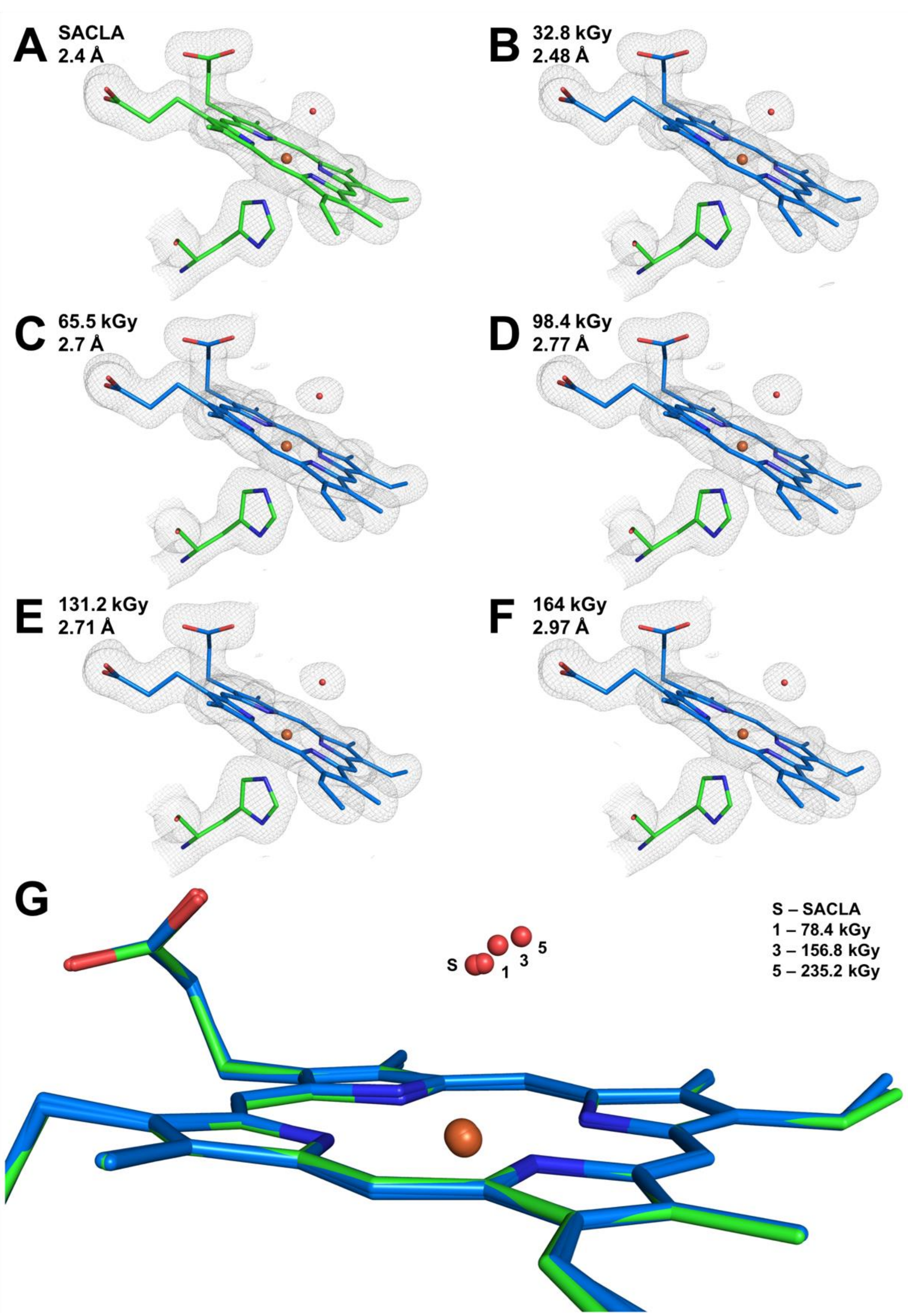

Figure S3 *2Fo-Fc* electron density maps contoured at 1σ for the haem environment of DtpAa in (A) the SFX dataset from SACLA, (B-F) selected structures from the low dose MSS-1 series; (G) Superposition of selected MSS-1 structures revealing the dose-dependent migration of the water molecule W1 away from the haem Fe.

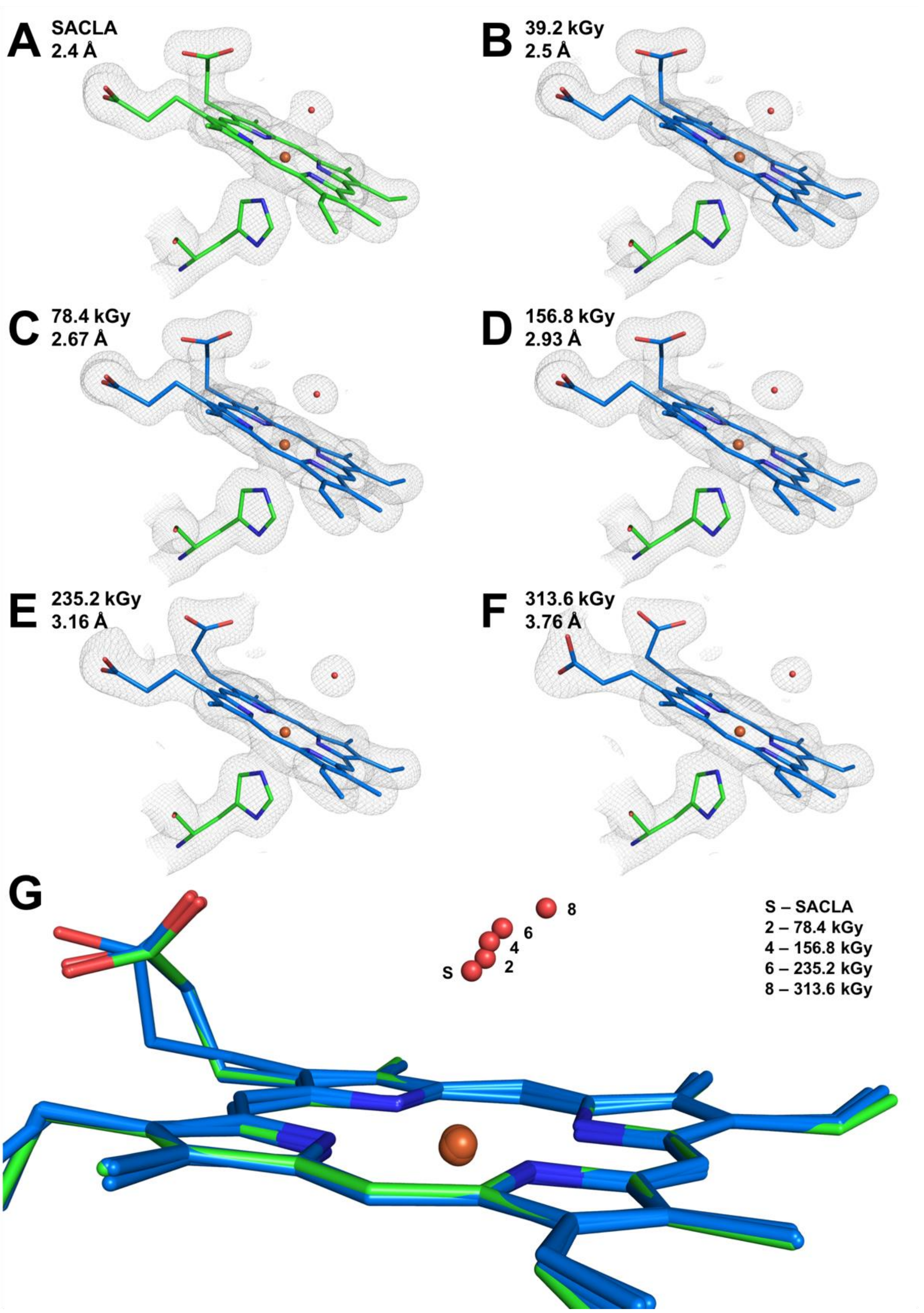

Figure S4 *2Fo-Fc* electron density maps contoured at 1σ for the haem environment of DtpAa in (A) the SFX dataset from SACLA, (B-F) selected structures from the higher dose MSS-2 series; (G) Superposition of selected MSS-2 structures revealing the dose-dependent migration of the water molecule W1 away from the haem Fe.

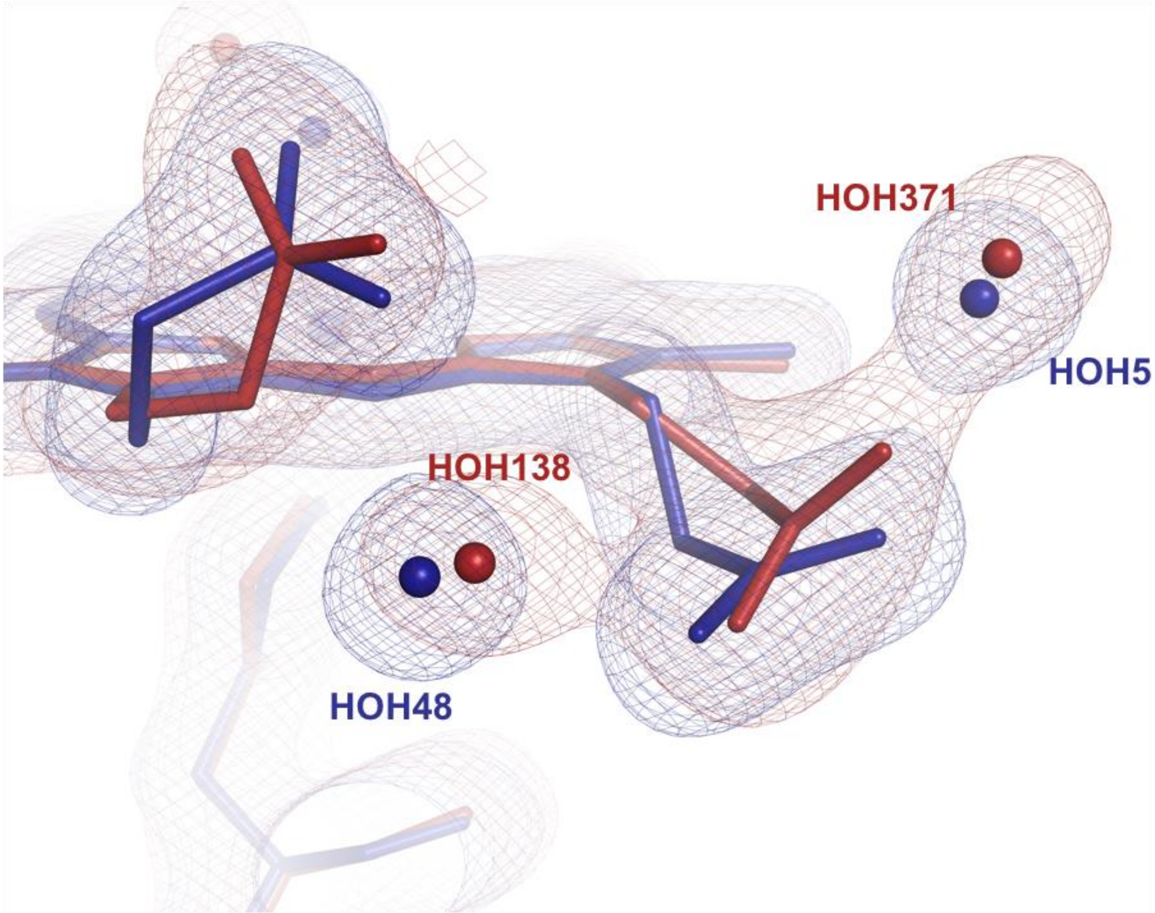

Figure S5 *2Fo-Fc* electron density maps contoured at 1σ for the haem propionate environment of DtpAa in the SFX dataset from SACLA (blue) and from the highest dose (313.6 kGy) structure from the MSS-2 series (red), showing corresponding waters between the 'damage free' and highest dose structure shift, a potential cause or effect of the flip in haem propionate(s).
